# Cross-cultural adaptation and psychometric properties of the Sinhala version of electronic health literacy scale: A cross-sectional validation study

**DOI:** 10.1371/journal.pone.0266515

**Published:** 2022-04-08

**Authors:** Sarath Rathnayake, Indrajith Prasanna Liyanage

**Affiliations:** 1 Department of Nursing, Faculty of Allied Health Sciences, University of Peradeniya, Peradeniya, Sri Lanka; 2 Department of Physiotherapy, Faculty of Allied Health Sciences, University of Peradeniya, Peradeniya, Sri Lanka; University of Defence in Belgrade, SERBIA

## Abstract

eHealth Literacy Scale (eHEALS) is the most widely used, valid and reliable tool to assess eHealth literacy skills, but no culturally appropriate tool exists to assess these skills among Sinhala-speaking Sri Lankans, including health professionals. This study aimed to cross-culturally adapt the original eHEALS from English to Sinhala and evaluate its psychometric properties. The study was conducted in two phases. The first phase involved translation and cross-cultural validation of the questionnaire. The second phase involved a cross-sectional survey conducted online among 268 health science students from a state university in Sri Lanka to confirm the psychometric properties of the questionnaire. An analysis of test-retest reliability was conducted with a subset of 72 students. The pre-final version of Sinhala eHEALS (Si-eHEALS) was developed following the cross-cultural adaptation process. The mean score of Si-eHEALS was 28.51±4.87. A satisfactory level of internal consistency was achieved (Cronbach’s alpha = 0.91). The test-retest reliability was acceptable (intraclass correlation coefficient -.776). Content validity index of Si-eHEALS was.97. The principal component analysis supported the unidimensionality of the scale, explaining 61.2 variance. There was a significant positive association between Si-eHEALS score with academic year (r_s_ = .146, p = .017), self-rated internet skills (r_s_ = .122, p = .046), usefulness of internet in health decision making (r_s_ = .212, p < .001) and importance of ability to access health resources on the internet (r_s_ = .230, p < .001), confirming concurrent validity. No significant difference based on gender (U = 5854, p = .550) and degree program (X^2^(2) = 2.965, p = .564) was found, confirming discriminant validity. In line with many previous validation studies, our study demonstrated good psychometric properties for Si-eHEALS. Si-eHEALS is a valid and reliable tool that assesses eHealth literacy in Sinhala speaking Sri Lankans, particularly health professionals.

## Introduction

Modern technologies are widely used in the healthcare industry. With the rapid spread of computer and Internet-based technologies, consumers are increasingly turning to online platforms for seeking health information [1]. Today, there are many information sources available online. The use of information and communication technologies (ICTs) for health is referred to as electronic health (eHealth) [2]. eHealth requires a skill set or literacy of its own, known as eHealth literacy [3]. eHealth literacy is defined as “the ability to seek, find, understand, and appraise health information from electronic sources and apply the knowledge gained to addressing or solving health problems” [3]. eHealth literacy consists of six core literacy skills: traditional literacy, health literacy, information literacy, scientific literacy, media literacy, and computer literacy [3]. These skills are essential for nurses and other health professionals because they facilitate their ability to find valid and reliable information about their practice. They can also assist their clients in finding reliable and accurate information on the internet [4, 5]. Moreover, eHealth literacy skills empower people by enabling them to participate in health decision-making [3] and maintaining a healthy lifestyle, good health status, and quality of life [6, 7]. Therefore, eHealth Literacy can be identified as an essential skill in modern healthcare.

However, the use of online information resources is often hindered by a variety of factors, such as physical access barriers, environmental barriers, and barriers related to resources and individual challenges and preferences [8]. Therefore, it is essential to determine consumers’ level of eHealth literacy to improve the skills needed and introduce eHealth interventions. The electronic health literacy scale (eHEALS) by Norman and Skinner [9] is a widely used, valid and reliable tool that assesses the individual’s perceived skills at finding, evaluating, and applying electronic health information to health problems. This assessment tool has been translated into different languages, for example, Arabic [10], Chinese [1], Dutch [11], Ethiopian [12], Hungarian [6], German [13], Iranian [14], Italian [15], Japanese [16], Korean [17], Norwegian [18], Spanish [19] and Sweden [20]. This tool has been used to assess eHealth literacy skills among diverse groups, such as the general public, students, health professionals, patients, and caregivers [5, 21–27]. Based on this scale, the different levels of eHealth literacy skills have been reported among health science students, including good [26, 27], moderate [5, 25] and inadequate levels [28].

In Sri Lanka, there is an increasing trend in using advanced ICTs, including the use of the internet and mobile phones [29] and the initiation of eHealth interventions [30]. The introduction of these activities is feasible due to the increased use of mobile technologies, dramatic improvement in accessibility, reduced cost of accessing, and advanced computer literacy in Sri Lanka [30]. However, Sri Lanka does not emphasize enough that eHealth literacy should be measured among health professionals and the general public. In Sri Lanka, two studies examined eHealth literacy skills among health science students (nursing) using the original version of eHEALS (English version) and found inadequate skills in eHealth literacy [28, 31]. One study identified the use of the English version as a limitation because students were more familiar with their mother tongue than with the second language, i.e., English [28]. Therefore, further investigation into eHealth literacy skills among health science students is crucial in the local context. Additionally, another study examined the validity and reliability of the original version of eHEALS using a sample of literate managers in the English language [32]. In this study, eHEALS has been demonstrated to be a reliable and valid tool for assessing eHealth literacy of English literate Sri Lankans, for example, managers. They have highlighted the need for developing eHEALS in local languages. To date, there is no eHealth literacy assessment tool in the local languages of Sri Lanka. Therefore, this study aimed to develop a culturally appropriate eHealth Literacy assessment tool in the Sinhala language, one of the official languages spoken by the majority of Sri Lankans. This pristine approach will enable clinicians and researchers to use the Sinhala Version of the eHEALS (Si-eHEALS) to assess eHealth literacy levels among Sinhala speaking Sri Lankans that will support planning eHealth interventions to promote the health and well-being of Sri Lankans.

## Objectives

The general objective of this study was to develop Si-eHEALS.

The specific objectives of this study were:

1. To cross-culturally adapt the original eHEALS from the English language to Sinhala.
2. To evaluate the psychometric properties, including reliability and validity of the Si-eHEALS.

## Materials and methods

### Research design

A cross-sectional validation study was conducted, consisting of two phases. Phase one involved the cross-cultural adaptation of the eHEALS. In phase two, a cross-sectional survey of health science students evaluated its psychometric properties. The assessment of validity (cross-cultural, content and construct validity) and reliability (internal consistency and test-retest reliability) of the instrument was carried out. Permission to use this scale in this study was obtained from the original developer (Dr. Cameron D. Norman).

### The instrument for validation: eHealth Literacy Scale

eHEALS assesses the individual’s perceived skills at finding, evaluating, and applying electronic health information to health problems [9]. This measure consists of eight items based on a five-point Likert scale ranging from one (strongly disagree) to five (strongly agree). A higher score of the eHEALS indicates higher eHealth literacy skills (total score range from 8 to 40). The internal consistency (Cronbach alpha reliability) in the original validation study was 0.88 [9]. Factor analysis has proven the construct validity and the unidimensionality of the scale [9]. Additionally, this measure consists of two questions that assess the perception of the internet as a tool to evaluate health information and make decisions about health.

### Phase 1: Cross-cultural adaptation process

The systematic method, consisting of five stages proposed by Beaton et al., was used [33].

#### Stage 1—Forward translation

Two independent bilingual translators who were fluent in English and Sinhala languages translated the English version of the questionnaire into Sinhala. The translators’ mother tongue was Sinhala. The issues they faced during the translation process were recorded independently. The translators reported difficulty in finding a suitable word for “health resources “in the Sinhala language. As a result, one forward translator has used the Sinhala term that represents “health information” while the other translator has used “health information (resources)” for “health resources” in the original version.

#### Stage 2—Synthesis of the forward translation

The researchers developed a common Sinhala version of the instrument (synthesis version) based on the forward translations and used the terms “health information (resources)” to represent “health resources” in the original version.

#### Stage 3—Backward translation

Two language experts who were fluent in English and Sinhala languages back-translated the synthesis version.

#### Stage 4—Expert committee review

A panel, including two health science lecturers, translators (forward and backwards) and researchers, reviewed the two forward translations and two backward translations with the original scale for semantic, idiomatic, experiential and conceptual equivalence [34]. A consensus was reached on the need for contextual changes primarily using simple language in Si- eHEALS. In Sinhala, the term “health resources” does not carry an accurate translation, and it is not widely used. This situation has been reported in several studies previously [6, 10, 18, 20], and this matter was also discussed with the original developer of eHEALS. As health resources include health information and related applications, the original developer agreed to use Sinhala words that mean “health information” or “health information and related applications” in the preliminary version of the Si-eHEALS. Finally, the term “health information and related applications” was selected.

*Content validity*. Additionally, the content validity index (CVI) was computed. When an instrument is considered content valid, it has a sufficient selection of items for measuring the construct being measured [35]. The preliminary version was distributed among a panel of experts, including one medical officer, one nursing officer, one university instructor in information technology, four lecturers in nursing (two-health informatics) and one lecturer in radiography for assessing the content validity index. The experts rated each item of the eHEALS based on the relevancy. A four-point Likert scale was used (1-not relevant, 2-somewhat relevant, 3- highly relevant, 4—strongly relevant).

*Content validity index*. To compute the content validity index for items (I-CVI), the number of experts rated as three or four was divided by the total number of experts [36]. The mean of total I-CVI was computed to assess the CVI of the scale (S-CVI) [36]. CVI >0.78 and 0.80 were considered adequate for I-CVI and S-CVI, respectively [36].

#### Stage 5—Pre-test

The preliminary version of the tool was pre-tested with eight health science students studying at the Faculty of Allied Health Sciences (FAHS), the University of Peradeniya, Sri Lanka. The pre-test helped to identify the clarity, readability and understandability of the items of the questionnaire. Three students reported difficulty in applying the correct meaning for “health information and related application”. Students agreed that the term “health information” is easier to understand and use than “health information and related application”. This matter was further discussed with the original developer of eHEALS. Finally, the Sinhala term representing “health information” was used in the final preliminary version of Si-eHEALS used in the psychometric evaluation. Similar to our study, the term “health information” has been used in Arabic [10] and Swedish [20] versions.

### Phase II—Psychometric evaluation

An online survey was conducted to evaluate the psychometric properties (validity and reliability) of the instruments.

#### Sample and setting

This study was conducted at the FAHS, University of Peradeniya, Sri Lanka. A convenience sample of health science students (medical laboratory sciences, nursing, pharmacy, physiotherapy and radiotherapy and radiography) from the first year to the fourth year enrolled on the bachelor’s degree programs participated in the study. In the faculty, 654 Sri Lankan students (mother tongue Sinhala—503 and mother tongue—Tamil 151) and one international student followed the undergraduate programmes. The students who did not give their consent and who were illiterate in Sinhala were excluded. A minimum desired sample size of 200 for exploratory factor analysis is recommended to obtain factor solutions [37]. A sub-sample of students participated in test-retest reliability. Although factor analysis requires a large sample size [38], test-retest reliability assessment requires small samples [3]. To achieve an intraclass coefficient (ICC) of.7 with two observations, a minimum sample size of 10 participants is required [39]. In this study, ICC was used in assessing test-retest reliability.

#### Instrument

Data were collected using a self-administered questionnaire through a google form, consisting of socio-demographic data, questions related to internet use, health-related internet use, and eHEALS. Socio-demographic characteristics included age, gender, degree program and educational level. As a measure of internet use, self-rated internet use skills (very poor, poor, average, good, very good) were used. To assess health-related internet use, two supplementary items in the eHEALS (“How useful do you feel the internet is in helping you in making decisions about your health?” and “How important is it for you to be able to access health resources on the internet?”) [9], were used. eHealth Literacy was evaluated by the preliminary version of Si-eHEALS developed in stage one.

#### Data collection

Data collection was conducted in May 2021. Necessary permission was obtained from the Dean, Faculty of Allied Health Sciences, University of Peradeniya. Invitation with the informed consent form and the online survey link was distributed among students through emails and social media groups (i.e., WhatsApp). Email addresses were collected from the faculty administration. Students who expressed their consent as part of the online survey completed the questionnaire. To assess the stability of the eHEALS, a sub-sample of students who expressed their willingness to participate in the primary survey, completed another wave of the survey after two weeks from the primary survey.

#### Data analysis

The data were downloaded to an excel datasheet, then to the Statistical Package for Social Sciences (SPSS) 22 version. There were no missing data. Descriptive statistics (number, percentage, mean and standard deviation) were used to characterize the sample and describe the eHEALS score. Similarly to previous studies [10, 12, 18, 20], reliability and validity were assessed as part of the assessment of Si-eHEALS psychometric properties [40]. In reliability testing, internal consistency and test-retest reliability were used. To establish construct validity, exploratory factor analysis (Principal component analysis), concurrent validity and discriminant validity were used. In addition to the computing CVI of the Si-eHEALS in phase I, similar to previous studies [10, 12, 20], floor and ceiling effects were run to confirm content validity based on the data from the cross-sectional survey [41]. The following section describes the tests used in the data analysis plan in this study.

*Internal consistency*. Internal consistency is a methodology that is commonly used with multi-item scales [42]. In this measure, each item of the scale is compared to all the others in order to assess how well it measures the concept [42]. Internal consistency of the eHEALS was evaluated by calculating Cronbach’s alpha scores [43]. Cronbach’s alpha value, 0.70 or greater, was accepted.

*Stability/Test-retest reliability*. Stability refers to the degree to which an instrument produces similar results over time and is measured by test-retest reliability [35]. Within two weeks after the first questionnaire delivery, a convenient sub-sample of students who expressed their willingness to participate in the second survey to determine the test-retest reliability completed the Si-eHEALS online. Test-retest reliability was estimated by the correlation between the scores at time one and at time two [43] with intraclass correlation coefficients (ICC) (ICC>0.7 was accepted) [41]. In test-retest analysis, intraclass correlation is the most widely used method [44].

*Construct validity*. The construct validity of an instrument involves the assessment of whether it actually measures the conceptual construct it claims to, which is determined by analysing how the conceptual and operational definitions of the variable fit together [42]. Factorability of the Si-eHEALS was determined by three methods: (1) computing inter-item correlations (correlation should be at least.3 with at least one other item), (2) the Kaiser-Meyer-Olkin (KMO) statistics of sampling adequacy (accepted value >.6), and (3) Bartlett’s test of sphericity (needs to be significant) [45]. Unidimensionality was established by performing the principal component analysis (PCA), following previously used methods [11, 15]. It was hypothesized that Si-eHEALS was unidimensional (eight items of the scale represent one construct). Factors with eigenvalues ≥ 1 were considered relevant for the test structure [6].

As a part of confirming construct validity, concurrent and discriminant validity was established through hypothesis testing. Concurrent validity is the ability of an individual’s score on an instrument or scale to provide an estimate of performance on another variable or criterion [42]. Discriminant validity establishes whether measures that are supposed to be unrelated are actually unrelated [46]. To test the concurrent validity, we hypothesized that there was a positive correlation between Si-eHEALS with the academic year, self-rated skills in using the internet and health-related internet use. The Si-eHEALS score was not normally distributed; therefore, the Spearman correlation test was used. We hypothesized that there was no significant difference in eHealth literacy based on gender and degree program to test the discriminant validity. Mann-Whitney U and Kruskal-Wallis H tests were used for group comparison. An Alpha level of significance of 0.05 was used.

*Floor and ceiling effects*. Floor and ceiling effects were evaluated and considered to be present if >15% of participants achieved the lowest or highest possible score [41]. The presence of floor and ceiling effects indicate that extreme items are missing in the lower or upper end of the scale, causing limited content validity of the tool [41].

#### Ethical considerations

Ethical approval for this study was obtained from the Faculty of Allied Health Sciences, University of Peradeniya, Sri Lanka. The information sheet was distributed with the online survey invitation. Consent was sought as a part of the online survey. In the online survey, participants filled out a section titled “Informed Consent” to indicate their consent. All data collection forms were given a code number, and coded information was used in data management.

## Results

### Socio-demographic characteristics of the study participants

In the main study, 268 students responded (78% female, mean age-24.22 ±1.29). A sub-sample of 72 students participated in the test-retest reliability testing. Table 1 presents the socio-demographic characteristics of the participants.

**Table 1:** Characteristics of the participants.

Characteristics		Study sample (N = 268) n (%)	Sub-sample for test-retest reliability (N = 72) n (%)
**Age (in years)**	mean = 24.22 *±* 1.29		mean = 24.58 ± 1.38
**Gender**	Male	59 (22)	22 (30.6)
	Female	209 (78)	50 (69.4)
**Degree program**	Medical Laboratory Science	30 (11.2)	4 (5.6)
	Nursing	103 (38.4)	33 (45.8)
	Pharmacy	40 (14.9)	11 (15.3)
	Physiotherapy	64 (23.9)	21 (29.2)
	Radiography and radiotherapy	31 (11.6)	3 (4.2)
**Academic year**	First	78 (29.1)	12 (16.7)
	Second	71 (26.5)	16 (22.2)
	Third	64 (23.9)	19 (26.4)
	Fourth	55 (20.5)	25 (34.7)
**Self-rated skills in using the internet**	Very poor	0	0
	Poor	11 (4.1)	0
	Average	125 (46.6)	22 (30.6)
	Good	111 (41.4)	44 (61.1)
	Very good	21 (7.8)	6 (8.3)
**Usefulness of internet in making decision about health**	Not useful at all	0	0
	Not useful	2 (0.7)	0
	Unsure	10 (3.7)	1 (1.4)
	Useful	202 (75.4)	56 (77.8)
	Very useful	54 920.1)	15 (20.8)
**Importance of ability to access health information on the internet**	Not important at all	1(0.4)	0
	Not important	1(0.4)	0
	Unsure	10 (3.7)	0
	Important	188 (70.1)	47 (65.3)
	Very important	68 (25.4)	25 (34.7)

### Distribution of Si-eHEALS score

The individual items and the overall score were not normally distributed and had negative skewness. The mean and median Si-eHEALS scores were 28.51±4.87 and 30, respectively. Table 2 presents mean, median, maximum and minimum values and skewness of items and total score. Based on the additional two questions of Si-eHEALS, the majority agreed that internet was useful in making decision towards their health (75.2%) and ability to access health resources on the internet was important (70.1%) (Table 1).

**Table 2:** Descriptive statistics and inter-item correlation of Si-eHEALS (n = 268).

Item	Mean (*SD*)	Median	Minimum, Maximum	Skewness
**1**	3.60±0.81	4	1,5	-1.635
**2**	3.57±0.79	4	1,5	-1.350
**3**	3.66±0.74	4	1,5	-1.786
**4**	3.69±0.76	4	1,5	-1.880
**5**	3.81±0.86	4	1,5	-2.300
**6**	3.50±0.68	4	1,5	-.874
**7**	3.27±0.76	4	1,5	-.766
**8**	3.40±0.87	4	1,5	-.777
**Total score**	28.51±4.87	30	8,39	-1.719

### Reliability testing

The internal consistency of Si-eHEALS (Cronbach’s alpha) was 0.908. Table 3 presents Cronbach’s alpha if an item deleted. The ICC between the total Si-eHEALS scores at time one and time two administration was 0.776.

**Table 3:** Cronbach’s alpha if an item deleted (n = 268).

Item	Item description	Cronbach’s alpha if an item deleted
**Item 1**	I know what health information are available on the internet	.899
**Item 2**	I know where to find helpful health information on the internet	.889
**Item 3**	I know how to find helpful health information on the internet	.889
**Item 4**	I know how to use the internet to answer my questions about health	.892
**Item 5**	I know how to use the health information I find on the internet to help me	.890
**Item 6**	I have the skills I need to evaluate the health information I find on the internet	.897
**Item 7**	I can tell high-quality health resources from low-quality health information on the internet	.897
**Item 8**	I feel confident in using information from the internet to make health decisions	.902

### Validity testing

#### Cross cultural validity

In the phase one, cross-cultural validity was established following the Beaton et al approach [33]. This process helped to establish semantic, idiomatic, experiential and conceptual equivalence between original English version and Sinhala version [33, 34] and to finalize the preliminary version of Si-eHEALS that underwent pre-test and further validation.

#### Content validity

For the item one and two in Si-eHEALS, CVI was 0.88, and for other six items, CVI was one. S-CVI was 0.97.

#### Construct validity

All the items of Si-eHEALS showed an inter-item correlation of at least.37 with at least one other item (Table 4). Kaiser-Meyer-Olkin Measure of Sampling Adequacy was.89, indicating a very good value. Bartlett’s Test of Sphericity was significant ((χ2 = 1328.12, P < .001), indicating the correlations between the items were significantly different from zero. PCA supported unidimensionality of the Si-eHEALS (eigenvalue = 4.9, 61.2% of variance explained). The first component showed a five times larger eigenvalue (4.9) than the second component’s eigenvalue (.92). Table 4 presents communalities and factor loading. The communalities of 8 items varied from 0.392 to 0.903. Factor loadings were high on this component and ranged from 0.626 to 0.950.

**Table 4:** Factor analysis and inter-item correlation.

Item	Communalities	Factor loading	Inter-item correlation							
**1**	.544	.738	1.							
**2**	.691	.831	.612	1						
**3**	.694	.833	.607	.808	1					
**4**	.666	.816	.551	.667	.713	1				
**5**	.694	.833	.575	.599	.602	.679	1			
**6**	.562	.749	.455	.449	.464	.558	.646	1		
**7**	.556	.746	.447	.506	.455	.472	.603	.604	1	
**8**	.495	.703	.372	.515	.510	.417	.482	.546	.626	1

Results of the Spearman correlation showed that there was a significant positive and weak association, between the Si-eHEALS score with academic year (r_s_ = .146, p = .017), self-rated internet skills (r_s_ = .122, p = .046) [47]. Moreover, we found a significant positive and fair relationship between the Si-eHEALS score with the usefulness of the internet in helping health decision making (r_s_ = .212, p >.001) and the importance of the ability to access health resources on the internet (r_s_ = .230, p >.001) [47]. Based on the Mann-Whitney U test, there was no significant difference in the Si-eHEALS score of male and female students (U = 5854, p = .550). Kruskal-Wallis H Test showed that there was no significant difference in Si-eHEALS score based on degree program (X^2^(2) = 2.965, p = .564).

#### Floor and ceiling effects

No floor and ceiling effects were found (two participants reported the worst possible score, and no participants reported the best possible score). Adequate floor and ceiling effect was >15% of the participants’ scores of the worst or the best possible score on the eHEALS [41].

## Discussion and conclusion

### Discussion

To the authors’ knowledge, this is the first reported study that translates and cross-culturally adapts the full version of eHEALS into the Sinhala language and tests its psychometric properties. For the cross-cultural adaptation process, we followed the approach recommended by Beaton et al. [33]. Similar approach has been used in the development of the Norwegian Version [18]. In cross-cultural adaptation of eHEALS, other methods followed were forward translation [13], forward and backward translation [1], forward and backward translation with expert committee review [6, 17, 20], World Health Organization guidelines [11, 15], Consensus-Based Standards for the Selection of Health Measurement Instruments (COSMIN) [10] and Kaiser and Steinmetz study protocol [12]. The five steps Beaton et al [33] approach that we followed in the present study was a systematic approach that incorporated forward and backward translation with several expert committee reviews and pre-testing with study participants. This approach led to confirm semantic, idiomatic, experiential, and conceptual equivalence between the source (English) and target (Sinhala) questionnaire [33, 34]. Therefore, this approach enabled researchers to develop a simple and culturally appropriate eHEALS in Sinhala language.

Similar to previous translation and validation studies of eHEALS [1, 6, 10, 12, 14–17, 20], the findings of this study support adequate psychometric properties, confirming Si-eHEALS is a valid and reliable tool to assess eHealth literacy skills among health science students. In contrast, two studies that examined psychometric properties indicated a need for further validation [18] or developing self-report instruments with high correlations with people’s actual eHealth literacy skills [11]. Recently, Gunasekara and Fernando have validated eHEALS using managers and senior working-age employees who were fluent in English in Sri Lanka [32]. One of the significant limitations in this study was that the original version of eHEALS was used without translation into a native language. Therefore, original eHEALS can be used for people fluent in the English language in Sri Lankan setting. By developing Si-eHEALS, the current study established cross-cultural and content validity, thus filling the above gap. Therefore, Si-eHEALS can be used to assess eHealth literacy skills of Sinhala speaking groups in Sri Lanka.

When using an assessment tool in other languages, translation and cross-cultural adaptation is essential. As recommended by Beaton et al., the present study followed five steps for cross-cultural adaptation [33] to ensure a culturally equivalent questionnaire translation [34]. Similar to previous studies that developed Arabic [10], Hungarian [6], Norwegian [18] and Swedish [20] versions of eHEALS, we faced a problem with choosing a proper word in Sinhala for the English concept of “health resource”; because it does not represent the same meaning and is not a widely used term in Sinhala. A similar situation has been reported in the above studies. Following the pre-test and after discussing with the original developer of eHEALS, the term “health information” was used in the final Si-eHEALS. Similar to the present study, the term “health information” has been used to represent “health resources” in Arabic [10] and Swedish [20] versions but “sources of health information” has been used in Hungarian [6] and Norwegian [18] versions. However, the structure of Si-eHEALS was equivalent to the original version that includes eight items with five points Likert Scale. Computation of CVI also showed high CVI for the items and whole scale, indicating strong content validity. As a result, we were able to develop a Si-eHEALS that is simple to read, understand and respond.

This study reported high Cronbach alpha coefficients, indicating very good internal consistency of Si-eHEALS. The test-retest analysis also showed acceptable stability. These findings were in line with previous validation studies, which reported very good internal consistency and [6, 11, 12, 14, 17] moderate to good test-retest reliability [6, 10, 12, 14, 17, 20]. Our study finding support Si-eHEALS is a reliable tool that gives the same results each time it is used in the same setting with the same type of subjects [48].

Norman and Skinner reported a single factor solution (unidimensionality) for the original version of eHEALS [9]. The present study also reported a one-factor structure for Si-eHEALS. A similar structure has been reported in previous translation and validation studies, for example, Arabic [10], Dutch [11], Hungarian [6] and Swedish [20]. Multi-dimensionality of eHEALS has been also reported in previous studies, for example, two factor solution (information seeking: items 1–5 & 8; information appraisal: items 6 & 7) [12, 13] and three-factor solution (awareness: items 1 and 2; skills: items 3–5; and evaluate: items 6–8) [18, 49]. Unidimensionality indicates that all the items of Si-eHEALS measure the single construct, i.e., eHealth literacy among health science students. This single one-factor structure helps to sum the item scores to a total score [20].

The present study reported acceptable concurrent and discriminant validity. As hypothesized, there was a significant positive association between Si-eHEALS score with the academic year, self-rated internet skills and health-related internet use (usefulness of internet in helping to make decisions about health and importance of the ability to access health resources on the internet), confirming the concurrent validity. Previous studies also identified an association between eHEALS score with education [10]. In line with findings of the present study, previous studies demonstrated a positive association with variables related to health information seeking, for example, health-related internet use [10, 18], computer literacy [1, 12], frequency of internet use [11], frequency of seeking health information and sources [6]. As we hypothesized, this study found that there was no significant difference in Si-eHEALS score based on gender and degree program, confirming the discriminant validity. Zrubka et al. also followed the examination of a possible association with socio-demographic data to establish discriminant validity which found no association between eHEALS with age, gender, education and income level in the general population [6].

In line with previous studies that developed Arabic [10], Dutch [11], Italian [15], Ethiopian [12], Norwegian [18] and Swedish [20] versions, this study reported acceptable floor and ceiling effect. Ideally, assessment tools should assess the entire spectrum of the phenomenon [20]. Terwee et al. state that if there are floor or ceiling effects, extreme items at the lower and upper ends of the measure are likely to be missing, indicating insufficient content validity [41]. In addition, if participants achieving the lowest or highest score cannot be distinguished, the scale will be less reliable [41]. Therefore, the acceptable floor and ceiling effect of the present study further support good content validity and reliability of the Si-eHEALS.

#### Limitations

In this validation study, convenience sampling was used in both main study and test-retest reliability. It may cause a bias because students interested in the internet use to seek information may participate. Nonetheless, an adequate number of students has been recruited for each study based on the sample size determination. Moreover, we recruited participants from a single university, adding to the study’s limitations.

## Conclusion

The Si-eHEALS has been successfully translated and culturally adapted for Sinhala speaking health sciences students in Sri Lanka. This research enabled us to develop a culturally appropriate tool for the assessment of eHealth literacy skills among health science students. Using simple and culturally appropriate language makes it easy to use. Our results supported adequate cross-cultural, content and construct validity, internal consistency and test-retest reliability. All statistical values of content validity, construct validity, internal consistency and test-retest reliability were acceptable. Therefore, Si-eHEALS is regarded as a valid and reliable tool for assessing eHealth literacy skills. In addition, the unidimensionality of the Si-eHEALS supports to measure of overall eHealth literacy level among participants. Therefore, Si-eHEALS is a valid and reliable tool for assessing eHealth literacy skills among Sinhala Speaking health professionals, particularly health science students. Health science teachers can use this tool to evaluate eHealth literacy and influencing factors of students to improve students’ online health information-seeking skills and update the curriculum. With further validation, Si-eHEALS can be used to assess eHealth literacy levels of other Sinhala speaking groups such as patients, caregivers and the public. This Sinhala version can be used to validate with different populations such as patients and caregivers. Besides Sinhala, Tamil is another official language in Sri Lanka. Therefore, the development of eHEALS in Tamil is recommended. eHealth literacy is an outcome variable for many eHealth interventions; therefore, clinicians and researchers would benefit from using this tool.

## Supporting information

S1 Checklist STROBE checklist. (DOCX)
